# Locked-in syndrome during stellate ganglion block

**DOI:** 10.4103/0019-5049.68376

**Published:** 2010

**Authors:** A Chaturvedi, HH Dash

**Affiliations:** Department of Neuroanaesthesiology, N.S. Centre, All India Institute of Medical Sciences (AIIMS), New Delhi, India

**Keywords:** Arterial injection, locked-in syndrome, stellate ganglion block

## Abstract

Intra-arterial injection of a local anaesthetic during stellate ganglion blockade may cause life-threatening complications. The usual complications are apnoea, unconsciousness and seizures. However, occasionally an unusual complication, ‘locked-in’ syndrome, has also been reported. In this syndrome the patients remain conscious despite their inability to move, breathe or speak. Here we describe a patient who developed features akin to the locked-in syndrome along with severe hypotension and bradycardia, after an injection of only 2 ml of lignocaine during a stellate ganglion block.

## INTRODUCTION

Stellate ganglion block (SGB) is frequently used for treatment of reflex sympathetic dystrophy (RSD) and causalgias of the upper limb.[1] Various complications of SGB include subarachnoid or epidural injection, recurrent laryngeal nerve block, seizures and blindness.[2–5] In addition, aphasia and haemiparesis due to central nervous system (CNS) toxicity, during SGB, has been described by Scott *et al*.[6] Dukes and Alexander have described a patient who developed apnoea, paralysis and had vertical eye movement during SGB, and termed it the transient locked-in syndrome.[7]

Our patient demonstrated symptoms similar to the locked-in syndrome along with cardiovascular depression (severe bradycardia and hypotension) during SGB, which have not been reported before.

## CASE REPORT

A 25-year-old male, weighing 62 kg, was a follow-up patient of brachial plexus injury of the right upper limb. He presented to our pain clinic with severe burning pain in the right forearm and hand. The pain was continuous and was graded 9/10 by the patient on the visual analogue scale (VAS) score. On examination there was no swelling, but discolouration of the skin of the forearm and hand was present. The hand was cold and gross wasting of the muscles of the hand and forearm was observed, but sensation to pinprick and fine touch was intact. The patient obtained no pain relief from medications such as analgesics, carbamazepine and antidepressants.

A right SGB was performed using the anterior paratracheal approach described by Carron and Litwiller.[8] The patient was placed supine with neck extended. The right anterior tubercle of the sixth cervical vertebra was palpated, while the carotid artery and sternocleidomastoid muscle were retracted laterally. A 22-gauge needle attached to a syringe containing 8 ml of plain lignocaine 1% (10 mg/ml) was inserted until its tip hit the transverse process just medial to the palpating finger. The needle was then withdrawn slightly and after negative aspiration, the drug was injected slowly with frequent aspiration in between.

After injecting about 2 ml of local anaesthetic (LA) lignocaine, the aspiration revealed a trickle of blood in the syringe and the needle was withdrawn. Within seconds the patient became apnoeic and was unable to follow commands to breathe, speak or move his limbs. He was immediately ventilated with 100% oxygen by facemask. The heart rate was 45 beats per minute and the systolic blood pressure 70 mm Hg. An intravenous (IV) line was secured. Atropine 0.6 mg, mephenteramin 6 mg and dexamethasone 8 mg were administered IV. One liter of Ringer’s lactate was infused rapidly. The haemodynamic parameters improved (H.R.70/min and B.P.110/70 mmHg), spontaneous respiration returned and the patient regained full consciousness within five minutes.

On asking the patient about the event he stated that he experienced flickering of stars in his eyes and tingling in ears. He had difficulty in breathing due to complete flaccidity of all muscles. He said that he was conscious and could hear, but was unable to speak or move his limbs in response to commands and he thought that he was going to die. After recovery he was observed for two hours in the recovery room and then discharged.

After four days a repeat right SGB was given using 8 ml of plain lignocaine 1%. As the patient had complication during the previous injection this time we gave the block under an X-ray image intensifier and haemodynamic monitoring, with frequent aspiration in between. Successful blockade (Horner’s syndrome and complete pain relief) was achieved and thereafter the patient received five more SGB with 8 ml of lignocaine 1%, on alternate days for long-term pain relief. The patient was followed up regularly, first weekly for a month and then every three months for two years. At the first follow-up the patient had a visual analogue score (VAS) of, 4/10 to 5/10, with mild hoarseness, which improved after two days. The patient was followed regularly and had pain relief for more than two years thereafter.

## DISCUSSION

Besides usual complications such as recurrent laryngeal block and brachial plexus block, the most serious complication of SGB is inadvertent intra-arterial injection of LA causing CNS toxicity. Two types of symptoms may occur: (i) excitatory (convulsive type) or (ii) depressive (circulatory type). Korevaar *et al*.[3] reported a case of generalised convulsion with transient unconsciousness after SGB, due to injection of bupivacaine into the vertebral artery. Others also reported similar excitatory CNS manifestations.[1,2,4] A case of blindness and aphasia without respiratory or haemodynamic depression, with complete recovery, was described by Sjeinfield *et al*.[5] Scott *et al*. reported a case of transient unconsciousness, apnoea, expressive aphasia and haemiparesis without haemodynamic changes after the injection of lignocaine into the carotid artery.[6]

Locked-in syndrome, which usually results from a lesion of the central and or ventral pons, is characterised by quadriplegia and an inability to produce speech. The reticular activating system is spared, thus consciousness is preserved. In this syndrome, selective supranuclear motor blockade in the brain stem resulted in motor paralysis and lower cranial nerve block, sparing eye blinking and vertical eye movements.[7] Usually the locked-in syndrome is caused by a cerebrovascular accident of the brain stem and carries high mortality and morbidity.[7] Reversible locked-in syndrome after basilar artery spasm has also been reported.[9]

Transient Locked-in syndrome during SGB was first reported by Dukes and Alexander.[7] Their patient remained conscious and was able to blink her eyes despite becoming apnoeic and quadriparetic. Recently, Tuz *et al*.[10] also described a case with transient locked-in syndrome, following SGB. In their case, despite negative aspiration, the injection prilocaine resulted in respiratory arrest and quadriplegia. However, the presence of haemodynamic stability and eyelid movement were suggestive of a transient locked-in syndrome. Our patient demonstrated symptoms akin to the transient reversible locked-in syndrome and became apnoeic and quadriparetic. Along with this he also developed severe bradycardia and hypotension immediately following the injection of only 2 ml of lignocaine 1%. He was not responding to verbal commands, but vertical eye movements were present. However, he became fully conscious and completely recovered within five minutes after resuscitation.

In our case, aspiration of blood was noted after 2 ml LA had been injected. The occurrence of immediate and short lasting symptoms favours intra-arterial injection as the cause. We re-emphasize that negative aspiration before injection is not a reliable indicator of correct needle placement.[3,10] As the carotid pulse was palpable all the time, the injection possibly was given into the vertebral artery. Various studies showed that cerebral blood volume was replaced after every two seconds, therefore, with rapid intra-arterial injection in the vertebral artery, lignocaine would have a bolus-like effect on the CNS, resulting in brain stem toxicity.[3] Korevaar *et al*. reported that the estimated minimum toxic dose of local anaesthetic injected into vertebral artery is only 4% of the minimum toxic IV dose. Therefore, the minimum toxic intra-arterial dose of lignocaine is 14 mg for a 60 kg patient.[3] In the present case the dose of 20 mg, was approximately one-and-a-half times that of the estimated intra-arterial toxic dose. The larger dose could be the reason for the depressive instead of excitatory CNS manifestations.

Serial SGB with LA has been recommended for long-term pain relief.[2] Neurolytic block with phenol,[1,2] and recently radiofrequency lesioning (RF) of the stellate ganglion, has also been used for long-term pain relief.[11] We normally use serial SGB with LA (Lignocaine or Bupivacaine). Most of our patients are getting long-term pain relief following serial injection with LA, with few minor and short-lived side effects, such as, temporary hoarseness of voice and an unpleasant sensation due to Horner’s syndrome.

In summary the present case demonstrates that despite negative aspirations, intra-arterial injection of LA can occur during SGB and can result in the locked-in syndrome, with severe haemodynamic depression that can be life threatening. It also emphasizes the importance of using the IV line, cardiovascular monitoring, resuscitative equipments, test dose of LA and if possible, fluoroscopy or ultrasound guidance,[12] in difficult cases.
